# Transcriptomic and functional analysis of fibroid extracellular vesicles

**DOI:** 10.1042/CS20250814

**Published:** 2026-05-05

**Authors:** Tsai-Der Chuang, Abigail Wiseman, Gabriela Alfaro, Shawn Rysling, Nhu Ton, Daniel Baghdasarian, Omid Khorram

**Affiliations:** 1 The Lundquist Institute for Biomedical Innovation, Torrance, CA, U.S.A.; 2 Department of Obstetrics and Gynecology, Harbor-UCLA Medical Center, Torrance, CA, U.S.A.; 3 Department of Obstetrics and Gynecology, David Geffen School of Medicine at University of California, Los Angeles, Los Angeles, CA, U.S.A.

**Keywords:** Exosome, Fibroid, Leiomyoma, lncRNA, miRNA, piRNA

## Abstract

Exosomes were isolated from cultures of fibroid explants and matched myometrial explants, and their RNA cargo was analyzed by next-generation sequencing to profile both long and short RNA species. Fibroid-derived exosomes (Fib-EXO) expressed the canonical extracellular vesicle markers CD81 and CD63, and their size distribution (30–200 nm) was consistent with exosomal vesicles. The RNA cargo of Fib-EXO generally reflected that of its tissue of origin, although selective enrichment of specific transcripts, such as piR-1398740 and piR-333378, suggested active loading mechanisms. Long RNA sequencing identified differential expression of protein-coding genes and long noncoding RNAs (lncRNAs) involved in RNA binding, cytoplasmic translation, exosome pathways, and PI3K/AKT and focal adhesion signaling. qPCR validation confirmed increased IGF2, HOXA10, and decreased IGFBP6 mRNA expression in Fib-EXO. Among lncRNAs, MSC-AS1, PART1, and H19 were overexpressed in Fib-EXO. Small RNA sequencing revealed differential expression of multiple small noncoding RNA classes, including Piwi-interacting RNAs, miRNAs, snRNAs, snoRNAs, and tRNAs. KEGG analysis showed that miRNAs were primarily associated with PI3K/AKT signaling, proteoglycans in cancer, interleukin signaling, and transcriptional regulation. Functionally, Fib-EXO were internalized by myometrial cells and promoted their proliferation with no effects on apoptosis. Furthermore, Fib-EXO enhanced angiogenesis in human umbilical vein endothelial cells. Fib-EXO increased the expression of vimentin, EZH2, DNMT1, TGF-β3, and c-MYC, and phosphorylated p65 protein, reduced COL1A1 and COL3A1 expression, and decreased miR-133a, miR-29, and miR-200c while increasing miR-21 in cultured myometrial smooth muscle cells, mirroring changes observed in fibroids. These findings indicate that Fib-EXO reprogram myometrial cells toward a fibroid-like phenotype characterized by increased proliferation, inflammation, and fibrotic features, thus contributing to fibroid propagation.

## Introduction

Uterine fibroids are the most common benign gynecologic tumors, affecting up to 80% of women. Their growth is dependent on estrogen and progesterone [1] and they frequently cause abnormal uterine bleeding, pelvic pain, and infertility [2,3]. Although significant progress has been made, the cellular origin of fibroids and the mechanisms that drive their expansion remain incompletely understood. Our research has focused on defining the molecular pathways underlying dysregulated protein-coding gene expression, with particular emphasis on the regulatory roles of noncoding RNAs [4]. We and others have identified several key miRNAs and long noncoding RNAs (lncRNAs) that critically influence fibroid gene expression [4]. However, it is not known whether these aberrantly expressed regulatory RNAs within fibroids can modulate gene expression in adjacent myometrial tissue.

Extracellular vesicles (EVs) are established mediators of intercellular communication and are enriched in noncoding RNAs [5,6], with important roles in tumorigenesis [7–10], fibrosis [11–14], and reproduction [15]. Based on these observations, we hypothesized that the RNA cargo of fibroid-derived EVs may alter gene expression in the myometrium, promoting a shift toward a fibroid-like phenotype. In the present study, we employed a fibroid and matched myometrial explant culture system, a physiologic model that retains all resident cell types including immune cells, to comprehensively characterize the large (coding and lncRNAs) and small RNA transcriptomes of fibroid EVs compared with matched myometrial EVs. We further demonstrate that fibroid EVs are internalized by myometrial cells, stimulate their proliferation, and modulate myometrial gene expression. Finally, we show that fibroid-derived EV cargo enhances angiogenesis.

EVs are membrane-bound vesicles released by cells into the extracellular environment, where they function as key mediators of intercellular communication. EVs are broadly classified by size and biogenesis into exosomes (30–200 nm), which originate from the endosomal pathway; microvesicles (50–1000 nm), which bud directly from the plasma membrane; and apoptotic bodies (1–5 μm), which are generated during programmed cell death. The molecular cargo of EVs, comprising proteins, noncoding RNAs, DNA, lipids, and metabolites, is determined by the cell of origin and can profoundly influence gene expression and cellular behavior in recipient cells [16].

To date, with the exception of a single study that evaluated EVs derived from a transformed fibroid cell line, a non-physiologic model [17], no studies have provided a comprehensive characterization of both coding and noncoding RNA cargo in primary fibroid-derived EVs or examined their impact on gene expression in myometrial cells.

## Materials and methods

### Myometrium and leiomyoma tissues collection

Surgically resected intramural leiomyomas (3–5 cm in diameter) and matched myometrial samples (*n* = 55) were obtained from patients undergoing gynecologic procedures at Harbor-UCLA Medical Center. All protocols were approved by the Institutional Review Board of the Lundquist Institute (IRB #18CR-31752-01R), and written informed consent was secured from each participant. None of the women had received hormonal therapy for at least three months before tissue collection. Donors ranged in age from 30 to 52 years (mean ± SD: 44 ± 4.4 years). The study population comprised 2 Asian, 4 Caucasian, 21 African American, and 28 Hispanic participants. MED12 mutations were identified in 73% of patients (*n* = 40). Immediately after excision, tissue specimens were snap-frozen in liquid nitrogen and stored at cryogenic temperatures until further processing, following established procedures [18,19].

### Reagents and spheroid cell culture

Myometrial smooth muscle cells (MSMCs) were maintained in DMEM containing 10% fetal bovine serum and grown under standard culture conditions, with medium replaced every 2–3 days until the cells reached full confluence. Experiments were conducted using cells between passages 1 and 3. For spheroid generation, MSMCs were plated into six-well plates coated with 0.5% agarose at a density of 1.5 × 10^5^ cells per well and incubated for 48 h, during which they self-organized into spheroids ranging from approximately 50 to 250 μm in diameter [20]. Each experiment was performed independently at least six times using MSMCs isolated from different patient samples. All reagents and culture materials were sourced from Sigma–Aldrich (St. Louis, MO), Invitrogen (Carlsbad, CA), and Fisher Scientific (Atlanta, GA).

### Isolation of exosomes and treatment

Freshly excised fibroid and matched myometrial tissues were rinsed thoroughly in PBS and maintained in culture for 72 h in medium supplemented with exosome-depleted FBS. Exosomes were isolated from the conditioned medium using a sequential ultracentrifugation strategy adapted from Yu et al. [21]. In brief, culture supernatants underwent low-speed centrifugation steps at 300×***g*** for 10 min, 2000×***g*** for 10 min, and 10,000×***g*** for 30 min to remove cells and debris. The clarified supernatant was subsequently ultracentrifuged twice at 100,000×***g*** to pellet the exosomal fraction. Pellets were washed and resuspended in 1× PBS, then either frozen at −80°C or used immediately for downstream applications. Control vesicles were prepared from unconditioned culture medium processed identically through all centrifugation steps to produce medium-derived EVs. For functional assays, MSMCs were grown to approximately 80% confluence, washed with PBS, and exposed to the designated amounts of fibroid-derived exosomes (Fib-EXO) at a final concentration of 100 μg/ml or medium-derived EVs for 48 h. The dose of EVs was selected based on prior literature indicating that biologically effective concentrations in *in vitro* systems typically fall within the range of ∼30–200 μg EV protein [22]. To ensure robust and interpretable outcomes, we therefore selected 100 μg representing a commonly used intermediate dose shown to elicit measurable biological effects in multiple studies [22].

### Characterization of exosomes

Exosome size profiles were assessed through nanoparticle tracking analysis (NTA), which was carried out by Alpha Nano Tech (Durham, NC). To further confirm vesicle morphology, samples were examined by transmission electron microscopy (TEM). TEM images were captured using a JEOL 1200EX instrument housed in the Advanced Electron Microscopy Core Facility at the University of California, Los Angeles (UCLA). Exosome concentrations were standardized based on total protein content, quantified using the BCA assay.

### Exosome labeling

Fib-EXO (100 μg) were fluorescently tagged using the ExoSparkler Membrane Labeling Kit (Dojindo Molecular Technologies, Rockville, MD) following the manufacturer’s instructions. After a 30-min incubation at room temperature, the labeling mixture was centrifuged at 10,000 rpm for 10 min to remove excess dye. The supernatant was discarded, and the labeled exosome pellet was gently resuspended in PBS before being applied to MSMC spheroid cultures. Internalization of the fluorescently labeled exosomes by MSMCs was visualized using a Leica TCS SP8 confocal microscope.

### RNA sequencing and bioinformatic analysis

RNA was isolated from paired Myo-EXO and Fib-EXO samples using TRIzol reagent (Thermo Fisher Scientific, Waltham, MA). RNA yield and quality were evaluated with a NanoDrop 2000c spectrophotometer (Thermo Scientific, Wilmington, DE) and by electrophoretic profiling on an Agilent 2100 Bioanalyzer (Agilent Technologies, Santa Clara, CA) as described previously [23,24]. Only RNA preparations with integrity values (RIN ≥9) were advanced to library construction. Construction of sequencing libraries, RNA sequencing, and downstream analyses for coding RNAs, lncRNAs, and small noncoding RNAs (sncRNAs) were performed at the Technology Center for Genomics & Bioinformatics at UCLA. For library generation, total RNA from each sample was used, 1 μg for coding and lncRNA libraries and 500 ng for small RNA libraries. Strand-specific cDNA libraries were prepared using the TruSeq library preparation system (Illumina, San Diego, CA) following the manufacturer’s guidelines. Barcoded libraries were pooled and subjected to sequencing on an Illumina MiSeq platform in a 35-bp single-end format, yielding approximately 10 million reads per library with alignment efficiencies of 80%–90% [18]. Bioinformatic analysis included visualization by hierarchical clustering, TreeView mapping, volcano plots, and principal component analysis (PCA). Functional pathway enrichment was conducted using Flaski [25] and RNAenrich [26]. All datasets met predefined quality control parameters for differential expression analysis. The RNA sequencing data have been deposited in the Gene Expression Omnibus database under accession number GSE326749.

### RNA isolation and qPCR analysis

RNA was extracted from fibroid tissue, matched myometrium, Myo-EXO, Fib-EXO, and MSMC cultures using TRIzol reagent (Thermo Scientific, Waltham, MA). RNA quantity and purity were assessed with a NanoDrop 2000c spectrophotometer (Thermo Scientific), and RNA integrity was evaluated on an Agilent 2100 Bioanalyzer (Agilent Technologies, Santa Clara, CA), following the manufacturers’ recommended procedures as previously described [27]. For cDNA synthesis, 1 μg of total RNA was reverse transcribed using random hexamer primers in accordance with the protocol supplied by Applied Biosystems (Carlsbad, CA). Quantitative PCR analyses were performed using SYBR Gene Expression Master Mix (Applied Biosystems) on an Applied Biosystems 7500 Fast Real-Time PCR System. Expression levels of coding and noncoding RNAs were normalized to appropriate reference genes: RNU6-2 for tissue and MSMC samples; miR-423-5p for Myo-EXO and Fib-EXO small RNA analyses; FBXW2 for tissue and MSMC samples; and ACTB for Myo-EXO and Fib-EXO large RNA analyses. All qPCR reactions were run in triplicate. Relative transcript abundance was determined using the 2^–ΔΔCt^ method, and data were expressed as fold change compared with the designated control group. Primer sequences used in these assays (5′–3′) are listed in Supplementary Table S1.

### Immunoblotting

After 48 h of treatment with Fib-EXO at a final concentration of 100 μg/ml or medium-derived EVs, MSMCs were harvested and processed for protein extraction, followed by immunoblotting using established laboratory procedures [28,29]. Cells were harvested in RIPA lysis buffer (Boston BioProducts, Ashland, MA) supplemented with EDTA, EGTA, PMSF, and a complete protease inhibitor cocktail (Roche Diagnostics, Indianapolis, IN). Lysates were briefly sonicated and clarified by centrifugation at 14,000 rpm for 10 min at 4°C. Protein concentrations were quantified using the BCA™ assay (Thermo Scientific Pierce, Rockford, IL). Thirty micrograms of protein from each sample were mixed with SDS sample buffer, denatured, and resolved on SDS–polyacrylamide gels. Proteins were then transferred to nitrocellulose membranes. Membranes were blocked in TBS–Tween containing 5% milk and incubated with primary antibodies directed against COL1A1, COL3A1, FN1, α-SMA, vimentin, EZH2, TGFβ3, and c-MYC (Proteintech Group, Chicago, IL); DNMT1, CD81, and CD63 (Santa Cruz Biotechnology, Dallas, TX); and cleaved caspase-3, phospho-p65 (Ser563), and total p65 (Cell Signaling Technology, Danvers, MA). After washing in TBS with 0.1% Tween-20, membranes were incubated with appropriate HRP-conjugated secondary antibodies. Protein bands were visualized using SuperSignal West Pico chemiluminescent substrate (Thermo Scientific Pierce) and captured by digital imaging. For normalization, membranes were stripped and reprobed with GAPDH (Santa Cruz Biotechnology) or evaluated using Ponceau S staining. Densitometric analysis was performed using ImageJ (http://imagej.nih.gov/ij/), and band intensities were normalized to GAPDH or to the Ponceau S reference signal. Data are presented as mean ± SEM, expressed relative to the control condition set to 1.

### Cell proliferation assay

MSMCs were plated in 96-well plates at a density of 1000 cells per well and allowed to adhere for 48 h. Cells were then exposed to Fib-EXO at a final concentration of 100 μg/ml or to medium-derived EVs under standard culture conditions (37°C, 5% CO_2_) for an additional 48 h. Proliferation was assessed using an MTT-based colorimetric assay, and representative images of the cultures were captured. For the assay, MTT reagent (Sigma, Carlsbad, CA, U.S.A.) was added directly to the wells at a final concentration of 1 mg/ml and incubated for 1 h at 37°C. Following removal of the medium, the resulting formazan crystals were dissolved in dimethyl sulfoxide, and absorbance was measured at 570 nm with background correction at 630 nm. Each condition was analyzed in six technical replicates, and the experiment was repeated independently three times.

### Angiogenesis assay

Endothelial tube formation was assessed using a commercial angiogenesis assay kit (Cell Biolabs Inc., San Diego, CA) in accordance with the manufacturer’s instructions. Human umbilical vein endothelial cells (HUVECs; Lonza Bioscience, Vacaville, CA) were grown to approximately 80% confluence, then transferred onto extracellular matrix (ECM) gel coated 96-well plates at a density of 1.5 × 10^5^ cells per well. Immediately after seeding, cells were exposed to Fib-EXO at a final concentration of 100 μg/ml or to medium-derived EVs. Cultures were maintained for up to 18 h at 37°C in a 5% CO_2_ incubator. After incubation, endothelial networks were visualized using the staining reagent supplied in the kit, and images were captured at 4× magnification with an inverted microscope. For quantitative assessment, each well was divided into four predefined fields, and tube length as well as branching points were quantified using ImageJ software. Data represent mean values ± SEM from three independent experiments, each performed in triplicate.

### Statistical analysis

All quantitative data are presented as the mean ± SEM. Statistical analyses were performed using GraphPad Prism (GraphPad Software, San Diego, CA). The distribution of each dataset was evaluated with the Kolmogorov–Smirnov test. Because the variables did not conform to a normal distribution, nonparametric approaches were used. Comparisons between paired groups were assessed with the Wilcoxon matched-pairs signed-rank test. A *P*-value less than 0.05 was considered statistically significant.

## Results

### Characterization of myometrial/fibroid EV

**Figure 1:**
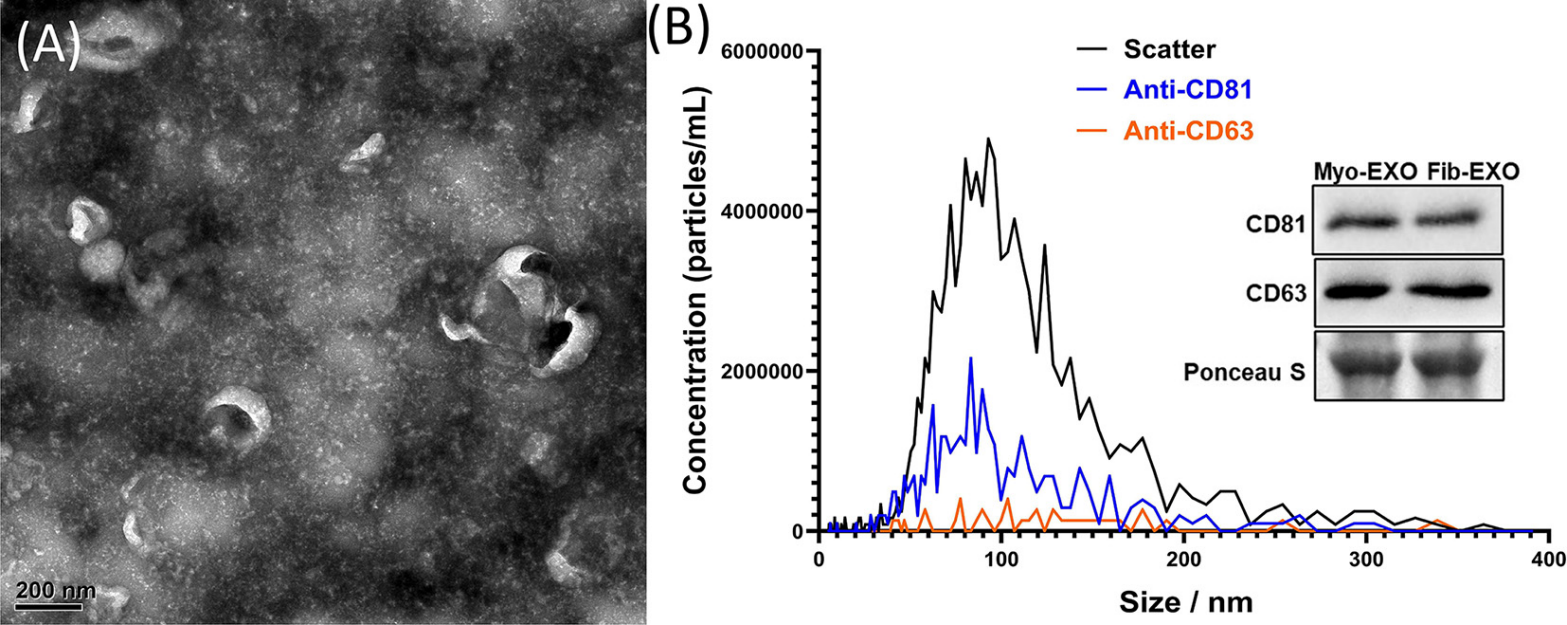
Characterization of exosomes isolated from human uterine fibroids. Characterization of exosomes isolated from human uterine fibroids. (**A**) TEM images reveal vesicles with the characteristic cup-shaped morphology typical of exosomes (scale bar: 200 nm; *n* = 3). (**B**) Fluorescent nanoparticle tracking analysis (f-NTA) shows a particle size distribution predominantly between 30 and 200 nm, consistent with the expected dimensions of exosomes (*n* = 3). (**C**) Immunoblot analysis demonstrates the presence of canonical exosomal tetraspanin markers CD81 and CD63 in exosomes derived from both fibroid tissue (Fib-EXO) and matched myometrium (Myo-EXO) (*n* = 3).

TEM demonstrated that EVs derived from fibroid and myometrial explants exhibit the characteristic cup-shaped morphology of exosomes (Figure 1A,B). NTA further confirmed the purity and size distribution of fibroid (Fib-EXO) and matched myometrial (Myo-EXO) EVs, which ranged from approximately 30 to 200 nm, consistent with exosomal vesicles (Figure 1B). Western blot analysis showed that fibroid/myometrial-derived EVs expressed the canonical exosomal tetraspanin markers, including CD81 (34%) and CD63 (4.7%) (Figure 1B).

### Transcriptomic analysis of fibroid/myometrial EVs

**Figure 2:**
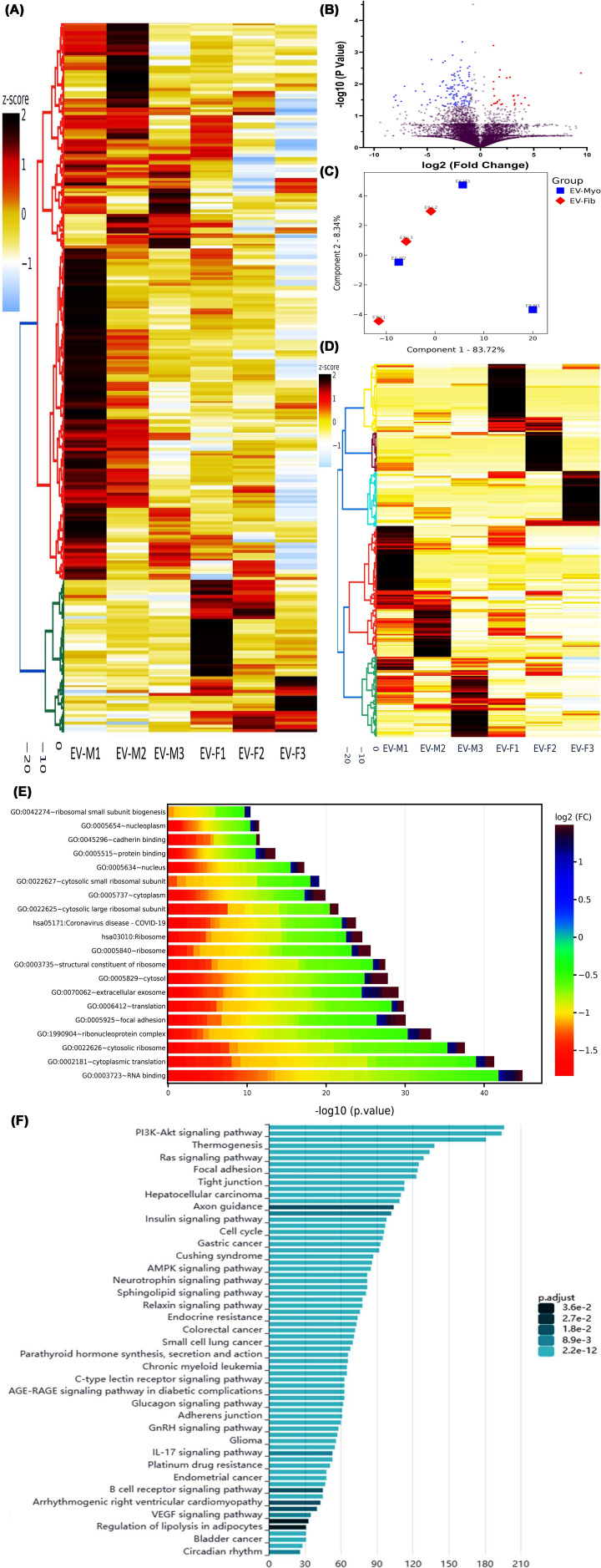
Transcriptomic profiling reveals distinct coding and noncoding RNA signatures in exosomes from fibroid tissue compared with matched myometrium. Transcriptomic profiling reveals distinct coding and noncoding RNA signatures in exosomes from fibroid tissue compared with matched myometrium. (**A**) Heatmap displaying hierarchical clustering of differentially expressed coding transcripts (fold change ≥2; *P* <0.05) from three paired exosome samples derived from fibroid (F) and myometrium (M). Gene expression values are shown as z-scores. (**B**) Volcano plot illustrating significantly up-regulated (red) and down-regulated (blue) coding RNAs, based on an FDR-adjusted *P*-value <0.05. (**C**) PCA demonstrating clear separation between fibroid-derived EVs (EV-Fib, red) and myometrium-derived EVs (EV-Myo, blue), with each point representing an individual sample. (**D**) Heatmap showing hierarchical clustering of differentially expressed lncRNAs (fold change ≥2; *P* <0.05) across the same paired EV samples, presented as z-score values. KEGG pathway and GO enrichment analyses for coding RNAs (**E**) and lncRNAs (**F**), highlighting biological processes and signaling pathways associated with the differentially expressed transcripts. Color scales represent log_2_ fold-change values converted into z-scores.

Previous studies have demonstrated that exosomes can transfer a variety of genetic materials, including DNA, RNA, and miRNAs. To characterize the differential expression profiles of coding RNAs, lncRNAs, and sncRNAs in Fib-EXO and matched Myo-EXO, we performed next-generation RNA sequencing (NGS) on exosomal RNA isolated from three pairs of cultured fibroid and their matched myometrial explants. Following normalization, 5186 coding RNAs displayed altered expression. Of these, 58 transcripts were up-regulated and 214 were down-regulated by twofold or greater in Fib-EXO relative to paired Myo-EXO, as shown by hierarchical clustering and TreeView analyses (Figure 2A). Volcano plot filtering further identified 27 significantly up-regulated and 117 significantly down-regulated coding RNAs (fold change ≥2; *P* <0.05) (Figure 2B). PCA demonstrated clear separation between Fib-EXO and Myo-EXO, and k-means clustering confirmed high internal consistency of the dataset (Figure 2C). Analysis of lncRNA expression revealed 1764 differentially expressed lncRNAs between Fib-EXO and Myo-EXO, including 10 up-regulated and 32 down-regulated lncRNAs showing at least a twofold change. Hierarchical clustering and TreeView visualization further distinguished these lncRNAs into discrete expression groups (Figure 2D). Gene Ontology (GO) and KEGG pathway enrichment analyses of both coding RNAs (Figure 2E) and lncRNAs (Figure 2F) indicated predominant involvement in RNA binding, cytoplasmic translation, extracellular exosome pathways, and key signaling pathways such as PI3K/AKT and focal adhesion.

**Figure 3:**
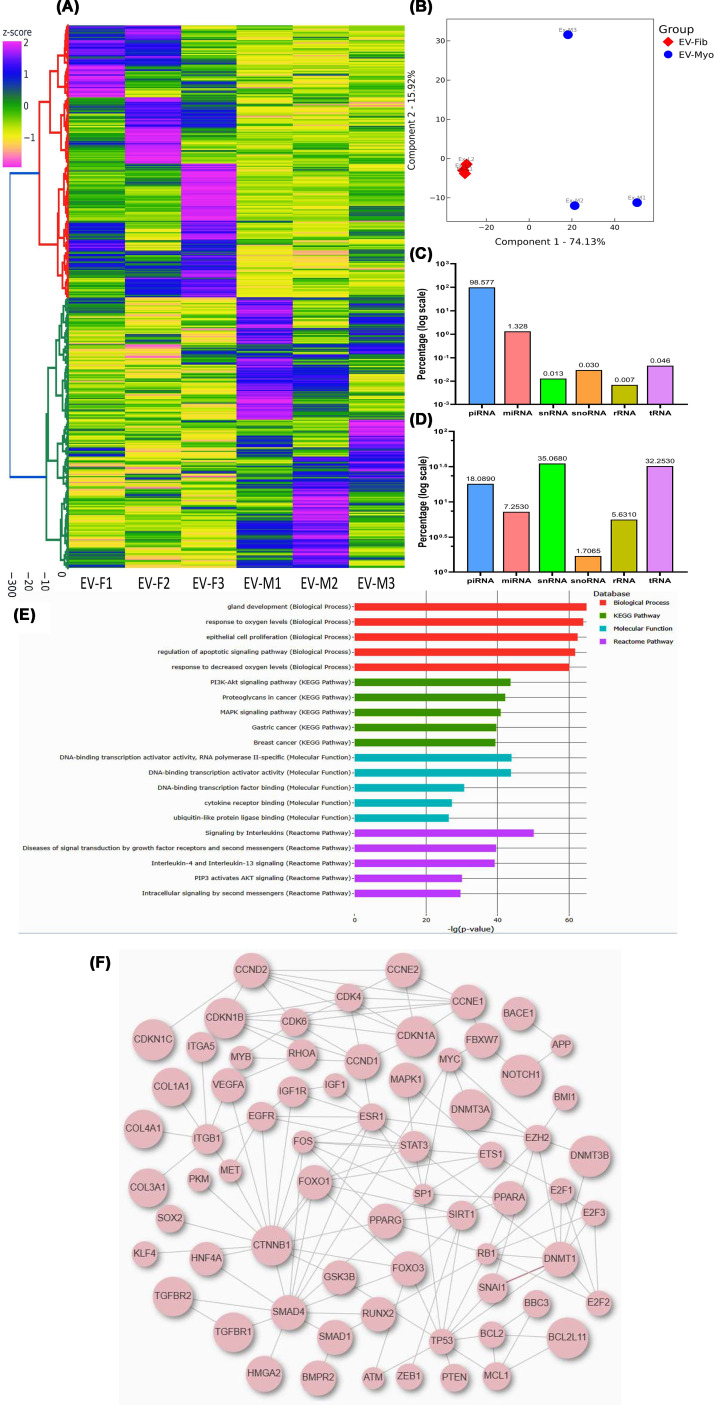
sncRNA landscape in exosomes derived from fibroid and matched myometrium. sncRNA landscape in exosomes derived from fibroid and matched myometrium. (**A**) Heatmap illustrating hierarchical clustering of differentially expressed sncRNAs (fold change ≥2; *P* <0.05) across three paired exosomal samples. Expression values are displayed as z-scores. (**B**) PCA demonstrating distinct clustering of fibroid-derived EVs (EV-Fib, red) and myometrium-derived EVs (EV-Myo, blue), with each point representing an individual sample. Distribution of sncRNA categories identified using two annotation platforms: AASRA (**C**) and miRMaster2 (**D**), revealing marked differences in classification proportions depending on the analytical database. (**E**) KEGG pathway and GO enrichment analyses of differentially expressed miRNAs, with color gradients reflecting log_2_ fold-change values (z-score normalized). (**F**) PPI network generated through RNAenrich (confidence cutoff = 0.9), illustrating regulatory connections associated with the miRNAs altered in Fib-EXO.

sncRNA sequencing revealed substantial differences in sncRNA expression profiles between Fib-EXO and matched Myo-EXO. Hierarchical clustering and TreeView analysis grouped these transcripts into distinct expression clusters (Figure 3A), while PCA demonstrated clear separation between Fib-EXO and Myo-EXO (Figure 3B). Using the AASRA database, we identified 30,351 differentially expressed sncRNAs, including 4 snRNAs, 9 snoRNAs, 403 miRNAs, 29,918 Piwi-interacting RNAs (piRNAs), 14 tRNAs, and 2 rRNAs (≥2-fold change, up- or down-regulated). AASRA-based categorization showed that Fib-EXO sncRNAs were predominantly piRNAs (98.577%), with smaller proportions of miRNAs (1.328%), tRNAs (0.046%), snoRNAs (0.03%), snRNAs (0.013%), and rRNAs (0.007%) (Figure 3C). In contrast, analysis of the same dataset using the miRMaster2 platform yielded markedly different sncRNA category distributions: 35.068% snRNAs, 1.707% snoRNAs, 7.253% miRNAs, 18.089% piRNAs, 32.253% tRNAs, and 5.631% rRNAs, with additional detection of differentially expressed circRNAs (Figure 3D). These discrepancies likely reflect differences between AASRA and miRMaster2 in database composition, alignment algorithms, multi-mapped read handling, and length-based filtering. Such variability is expected given the fragmented and heterogeneous nature of exosomal sncRNAs. Pathway enrichment analyses (KEGG, Gene Ontology Biological Process and Molecular Function, and Reactome Pathway) performed on the 238 miRNAs mapped by RNAenrich revealed predominant association with PI3K/AKT signaling, proteoglycans in cancer, interleukin signaling, and DNA-binding transcription activator activity (Figure 3E). A protein–protein interaction (PPI) network generated with a confidence threshold of 0.9 further demonstrated that these miRNA-associated targets form highly interconnected regulatory clusters and key signaling nodes (Figure 3F).

### PCR validation of key genes

**Figure 4:**
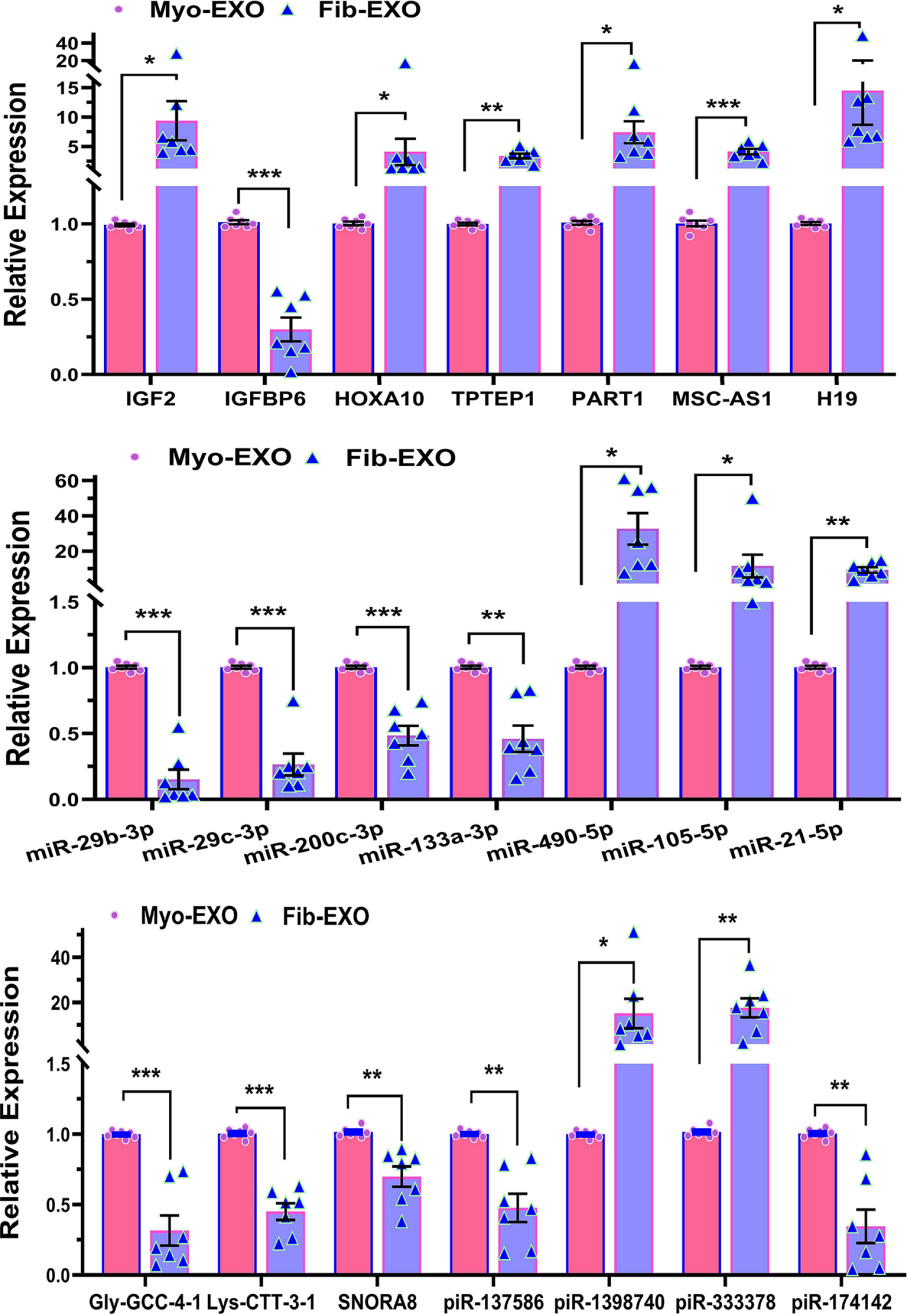
Validation of differentially expressed coding and noncoding RNAs in fibroid- versus myometrium-derived exosomes. Validation of differentially expressed coding and noncoding RNAs in fibroid- versus myometrium-derived exosomes. Quantitative RT-PCR was performed on seven paired exosome samples (Fib-EXO and Myo-EXO) to assess the expression of selected transcripts, including coding genes (IGF2, IGFBP6, HOXA10), lncRNAs (TPTEP1, PART1, MSC-AS1, H19), and a panel of sncRNAs (miR-29b-3p, miR-29c-3p, miR-200c-3p, miR-133a-3p, miR-490-5p, miR-105-5p, miR-21-5p, Gly-GCC-4-1, Lys-CTT-3-1, SNORA8, piR-137586, piR-1398740, piR-333378, and piR-174142). Data are shown as mean ± SEM. Statistical significance is indicated as *P* <0.05, *P* <0.01, and *P <0.001.

**Figure 5:**
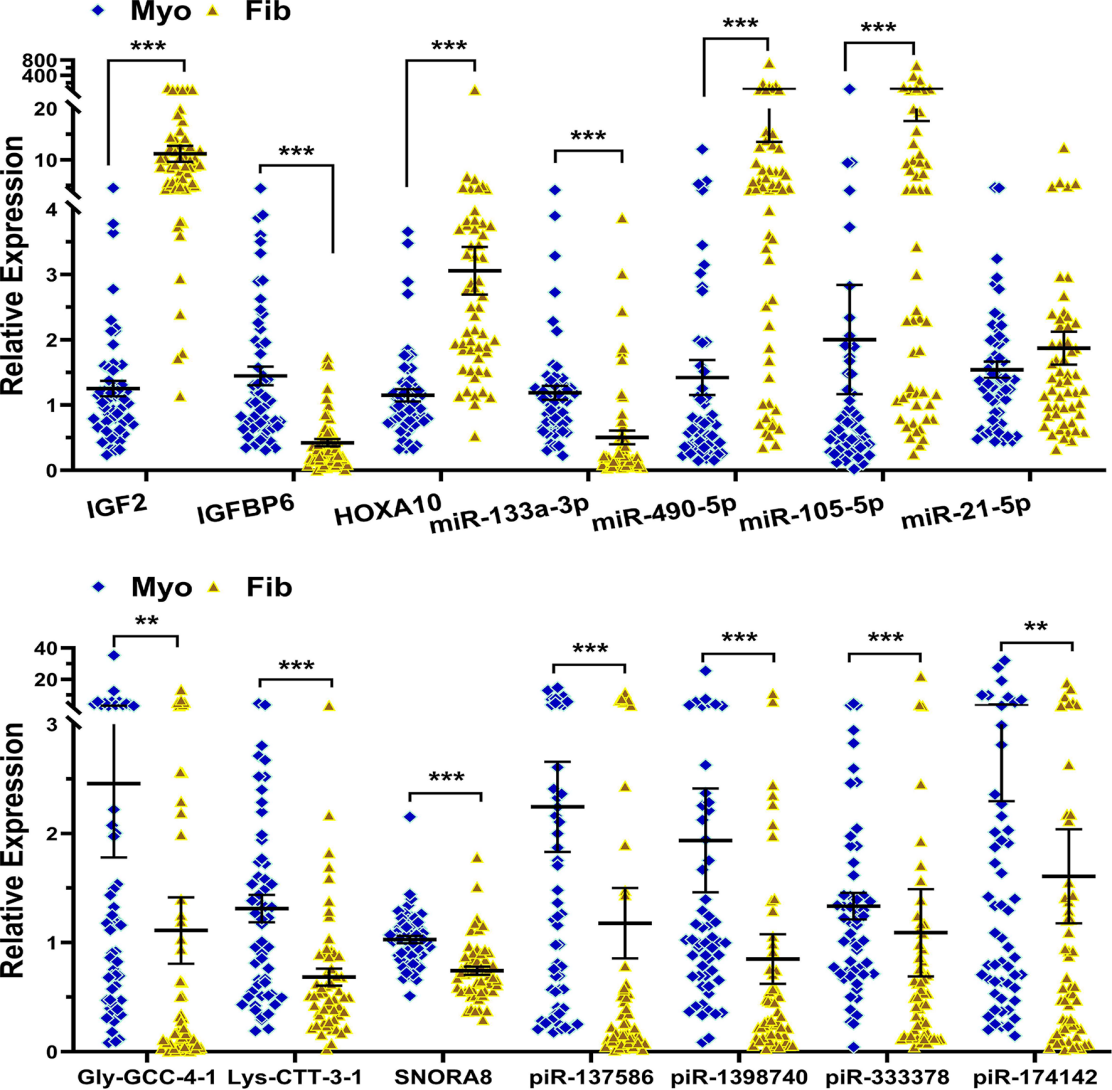
qRT-PCR validation of selected coding and sncRNAs in paired fibroid and myometrial tissues. qRT-PCR validation of selected coding and sncRNAs in paired fibroid and myometrial tissues. Expression levels of coding transcripts (IGF2, IGFBP6, HOXA10) and a panel of sncRNAs (miR-133a-3p, miR-490-5p, miR-105-5p, miR-21-5p, Gly-GCC-4-1, Lys-CTT-3-1, SNORA8, piR-137586, piR-1398740, piR-333378, and piR-174142) were quantified in 55 matched fibroid (Fib) and myometrium (Myo) samples using qRT-PCR. Values are expressed as mean ± SEM. Statistical significance is denoted as *P* <0.01 and *P <0.001.

We next selected 21 novel coding RNAs, lncRNAs, and sncRNAs identified from the EV NGS analysis and validated their expression by qRT-PCR in seven Fib-EXO samples compared with matched Myo-EXO. Among these transcripts, IGF2, HOXA10, TPTEP1, PART1, MSC-AS1, H19, miR-490-5p, miR-105-5p, miR-21-5p, piR-1398740, and piR-333378 were significantly up-regulated in Fib-EXO, whereas IGFBP6, miR-29b-3p, miR-29c-3p, miR-200c-3p, miR-133a-3p, Gly-GCC-4-1, Lys-CTT-3-1, SNORA8, piR-137586, and piR-174142 were significantly down-regulated (Figure 4). We further evaluated the expression of these validated transcripts in fibroid tissues and their matched myometrium (Figure 5; *n* = 55). The expression profiles of additional genes not shown in Figure 5 have been previously reported [19,30–33]. Comparison of Fib-EXO expression with tissue expression revealed that nearly all validated transcripts showed similar directional changes in both exosomes and their tissue of origin. An exception was miR-21-5p, which was overexpressed in Fib-EXO but not significantly altered in our tissue samples, in contrast with prior reports of its upregulation in fibroids [34]. Additionally, piR-1398740 and piR-333378 were up-regulated in Fib-EXO but down-regulated in fibroid tissue relative to myometrium, indicating that selective and active loading of these piRNAs occurs during exosome biogenesis in fibroids.

### Effect of fibroid EV on myometrial cell proliferation/apoptosis

**Figure 6:**
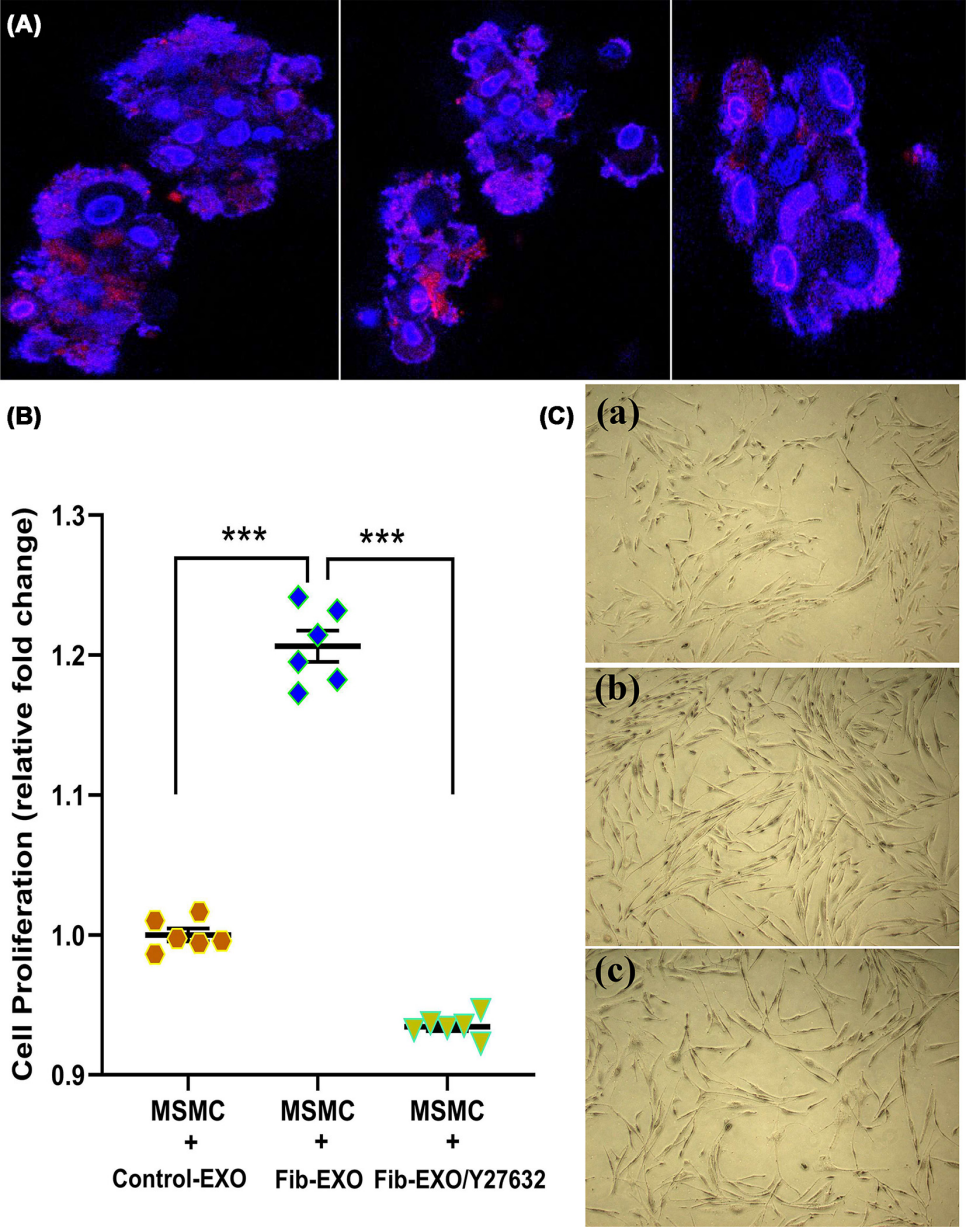
Uptake of Fib-EXO by MSMC spheroids and their effects on cellular proliferation. Uptake of Fib-EXO by MSMC spheroids and their effects on cellular proliferation. (**A**) Confocal microscopy images demonstrating internalization of red fluorescent-labeled exosomes by MSMC spheroids; nuclei are counterstained with DAPI (blue). Images represent multiple optical sections. (**B**) MTT analysis showing that Fib-EXO markedly enhanced MSMC proliferation, an effect that was abolished by treatment with the EV uptake inhibitor Y27632 (10 μM). Medium-derived EVs (Control-EXO) served as a negative control. Data (*n* = 6) are reported as mean ± SEM, with *P <0.01 indicating significant differences between groups. (**C**) Representative brightfield micrographs illustrating differences in MSMC morphology and density following treatment: (**a**) MSMCs incubated with Control-EXO; (**b**) MSMCs treated with Fib-EXO; and (**c**) MSMCs exposed to Fib-EXO in the presence of Y27632 (10 μM).

Exosomes function in both autocrine and paracrine capacities, transmitting molecular signals to nearby and distant cells to modulate their behavior. We hypothesized that Fib-EXO may influence the uterine microenvironment, particularly the myometrium, by delivering fibroid-derived bioactive factors. To test this, we co-cultured MSMC spheroids with fluorescently labeled Fib-EXO and assessed exosome uptake using confocal microscopy. Fib-EXO were readily internalized by MSMC spheroids after 24 h of co-culture (Figure 6A), thus providing the basis to influence gene expression programs governing proliferation and angiogenesis.

We next evaluated the proliferative response of MSMC following exposure to Fib-EXO. MTT assays performed 48 h after treatment revealed a significant increase in MSMC proliferation in the Fib-EXO-treated group, an effect that was abolished by the EV uptake inhibitor Y27632 (Figure 6B). Representative photomicrographs further illustrated the cellular proliferative changes induced by Fib-EXO (Figure 6C).

### Angiogenic properties of fibroid EVs

**Figure 7:**
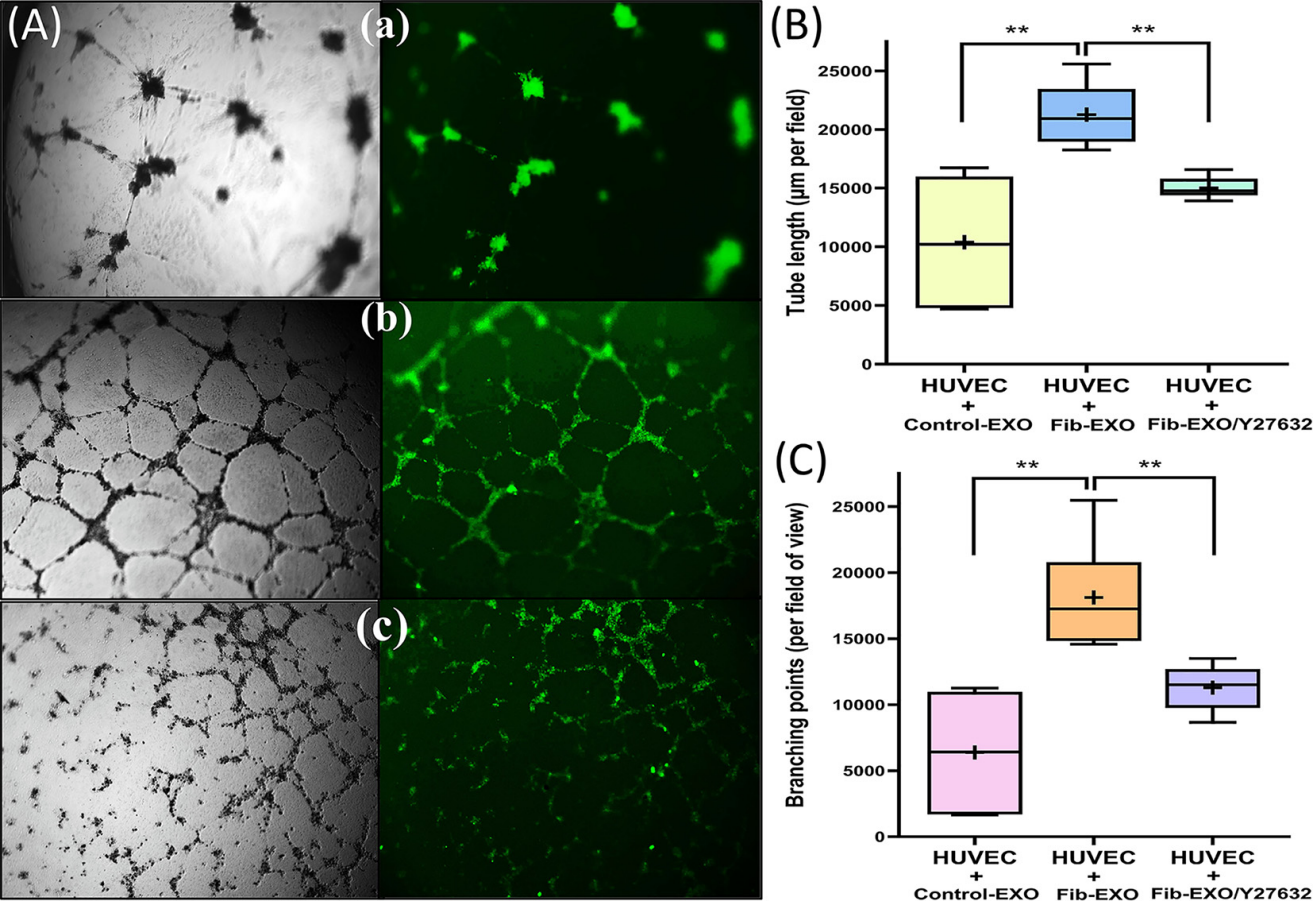
Fib-EXO enhance angiogenic activity in HUVECs. Fib-EXO enhance angiogenic activity in HUVECs. (**A**) Representative fluorescence images of HUVECs seeded on Matrigel and exposed to (**a**) medium-derived EVs (Control-EXO), (**b**) Fib-EXO, or (**c**) Fib-EXO in the presence of the EV uptake inhibitor Y27632 (10 μM; Fib-EXO/Y27632). Cells were stained using the reagent provided in the angiogenesis assay kit. Quantitative analysis of tube formation shows that HUVECs treated with Fib-EXO exhibited significantly increased total tube length (**B**) and total branching points (**C**) relative to Control-EXO or inhibitor-treated samples. Data are presented as *P* <0.01 (**).

To further assess the impact of Fib-EXO on the uterine microenvironment, we co-cultured HUVECs with Fib-EXO and quantified angiogenic responses, including tube formation, total tube length, and branching. HUVECs plated on Matrigel and treated with Fib-EXO exhibited a significant increase in tube formation and branching points compared with cells treated with medium-derived EVs. These pro-angiogenic effects were abolished by the EV uptake inhibitor Y27632 (Figure 7A–C), demonstrating that Fib-EXO stimulate angiogenesis in an uptake-dependent manner.

### Effect of fibroid EVs on myometrial gene expression

**Figure 8:**
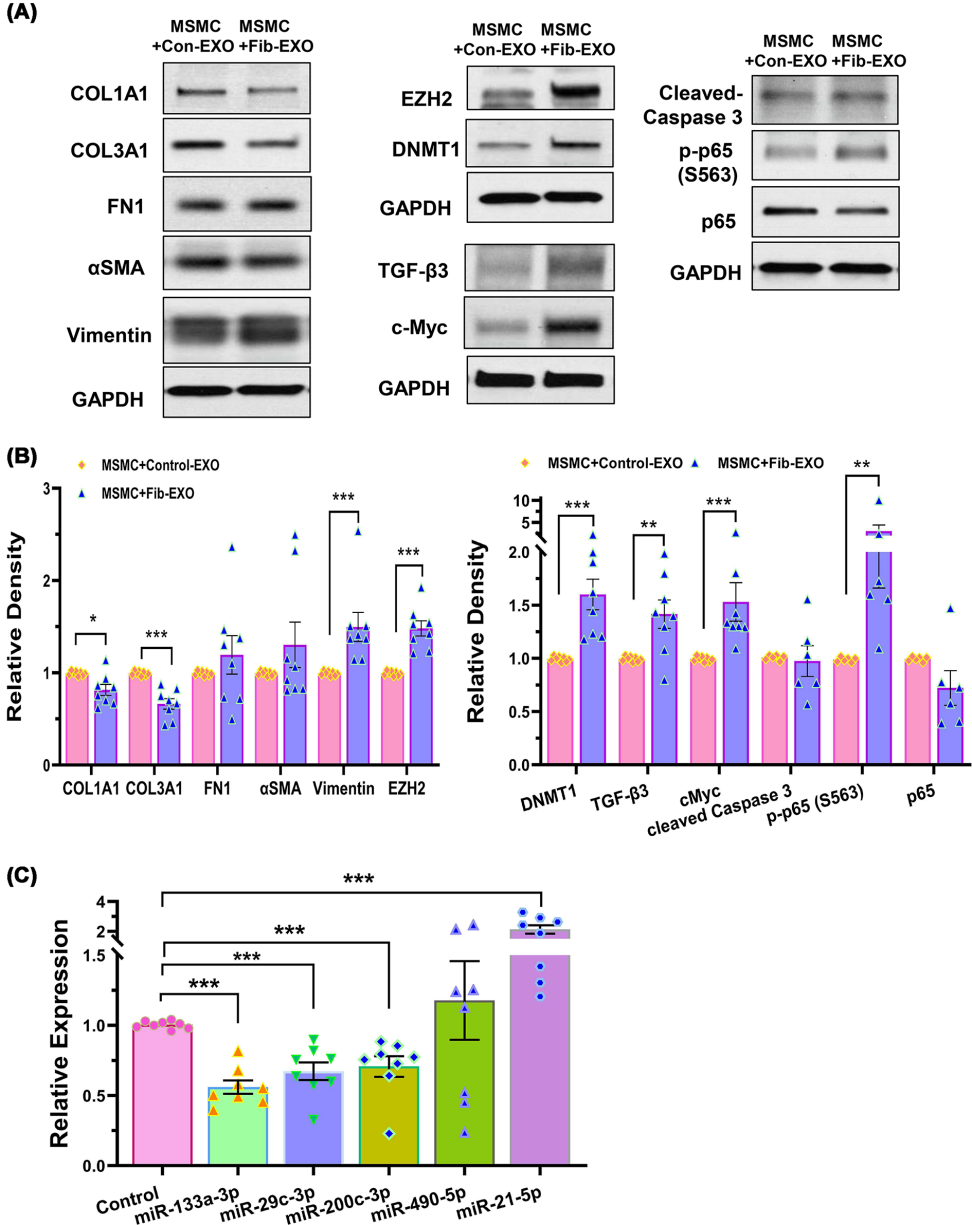
Fib-EXO alter protein and miRNA expression profiles in MSMCs. Fib-EXO alter protein and miRNA expression profiles in MSMCs. (**A**) MSMCs were exposed to Fib-EXO or medium-derived EVs (Con-EXO) for 48 h, after which protein lysates were analyzed by immunoblotting. Representative blots display the expression of COL1A1, COL3A1, FN1, α-SMA, vimentin, EZH2, DNMT1, TGF-β3, and c-MYC (*n* = 8), as well as cleaved caspase-3, phosphorylated p65 (Ser563), and total p65 (*n* = 6). (**B**) Corresponding quantitative analyses of band intensities are shown in bar graphs, normalized to internal controls. (**C**) Relative expression levels of selected miRNAs (miR-133a-3p, miR-29c-3p, miR-200c-3p, miR-490-5p, and miR-21-5p) in MSMCs treated with Fib-EXO or Con-EXO for 48 h (*n* = 8). Data are presented as mean ± SEM. Statistical significance is denoted as *P* <0.05, *P* <0.01, and *P <0.001.

We next examined the effects of Fib-EXO on myometrial gene expression. Following 48 h of treatment, MSMCs exposed to Fib-EXO showed significantly increased protein levels of vimentin, EZH2, DNMT1, c-MYC, TGF-β3, and p-p65 (S563), whereas COL1A1 and COL3A1 protein expression was decreased (Figure 8A,B). In contrast, no significant changes were observed in the protein levels of FN1, αSMA, cleaved caspase-3, and total p65. Fib-EXO also induced changes in miRNA levels in MSMC that mirrored the expression profiles observed in exosome and fibroid tissues, with the exception of miR-490-5p and miR-105-5p (Figure 8C). MSMCs treated with Fib-EXO had reduced expression of miR-133a-3p, miR-29c-3p, and miR-200c-3p, and increased expression of miR-21-5p. Although miR-105-5p was among the most highly up-regulated miRNAs in Fib-EXO, it was not detectable in MSMCs following Fib-EXO treatment. These findings indicate that Fib-EXO can modulate the expression of protein-coding and noncoding genes that are key regulators of cell proliferation, inflammation, epigenetic signaling, and ECM composition in myometrial cells.

## Discussion

The findings of the present study demonstrate that Fib-EXO express canonical EV protein markers, including the tetraspanins CD81 and CD63, and exhibit a size range of 30–200 nm, consistent with classification as exosomes. The RNA cargo of Fib-EXO largely mirrored that of the fibroid tissue of origin with some exceptions (piR-1398740 and piR-333378), suggesting an active loading mechanisms for select transcripts during exosome biogenesis. Long RNA sequencing revealed differential expression of multiple protein-coding genes and lncRNAs, with enrichment in pathways related to RNA binding, cytoplasmic translation, extracellular exosome function, and key signaling pathways such as PI3K/AKT and focal adhesion. qPCR validation confirmed increased expression of IGF2 and HOXA10 and decreased IGFBP6 mRNA in Fib-EXO compared with Myo-EXO, and elevated levels of the lncRNAs TPTEP1, PART1, and H19. Small RNA profiling showed that the relative proportions of sncRNA classes varied depending on the analytical database; nevertheless, both fibroid- and myometrium-derived exosomes contained abundant piRNAs, miRNAs, snRNAs, snoRNAs, and tRNAs, many of which were differentially expressed in Fib-EXO. KEGG analysis revealed that differentially expressed miRNAs were predominantly associated with PI3K/AKT signaling, proteoglycans in cancer, interleukin signaling, and transcriptional regulation. Functionally, Fib-EXO were internalized by myometrial cells, stimulated their proliferation, and exhibited pro-angiogenic effects in HUVECs. Fib-EXO treatment increased MSMC protein levels of vimentin, EZH2, DNMT1, TGF-β3, c-MYC, and p-p65 (S563) while reducing COL1A1 and COL3A1, with no significant effects on FN1 or α-SMA. Additionally, Fib-EXO decreased expression of miR-133a-3p, miR-29b-3p, miR-29c-3p, and miR-200c-3p in MSMC, consistent with expression patterns observed in fibroid tissue and fibroid exosomes. Collectively, these results indicate that Fib-EXO actively modulate myometrial gene expression, promoting a fibroid-like phenotype, and may serve as key vesicles driving fibroid propagation and growth.

NGS analysis revealed enrichment of genes associated with RNA binding, focal adhesion, and RNA polymerase complexes. Notably, a substantially greater number of protein-coding genes were down-regulated than up-regulated in Fib-EXO compared with Myo-EXO, suggesting that Fib-EXO may signal a reduction in specific protein expression within target myometrial cells. Many of the down-regulated transcripts were involved in chromatin remodeling (GATAD2B, BRD2, BPTF), immune regulation (CXCL5, CCL2), tumor suppression (KLF6, AHNAK, KLF9), ribosomal structure and assembly (RPL13A, RPL34, RPL22, RPL7A), cytoskeletal scaffolding (SPTBN1, TUBA1C, AKAP12, ACTG1), calcium homeostasis (S100A6, CALM3, C4orf3, EFCAB14), and protein translation (EIF4B, EIF3E, EIF1). The two most down-regulated genes, CCL2 and CXCL5, encode chemokines critical for immune cell recruitment, CCL2 for monocytes and macrophages [35], and CXCL5 for neutrophils [36]. Their suppression suggests that Fib-EXO may attenuate immune activation at the target site, thereby enabling unchecked proliferation of recipient myometrial cells and promoting immune evasion.

In contrast, many of the up-regulated transcripts in Fib-EXO were associated with mitochondrial function, ribosomal components, cytoskeletal elements, and vesicular transport. Mitochondrial genes included components of the electron transport chain (MT-CYB, MT-ND1, MT-ND2, MT-ND4, MT-CO1), ATP synthesis (MT-ATP6, MT-ATP8), and the mitochondrial ribosome (MT-RNR1, MT-RNR2). The overrepresentation of mitochondrial genes in Fib-EXO may reflect a mechanism for transferring mitochondrial components from fibroids to recipient myometrial cells, potentially enhancing their bioenergetic capacity to support increased proliferation and angiogenesis.

Fibroid- and myometrium-derived exosomes were found to be rich in noncoding RNAs, including long RNA transcripts (lncRNAs and circRNAs) and various classes of small RNAs (piRNAs, tRNAs, snoRNAs, snRNAs, rRNAs). The distribution of sncRNA categories differed substantially depending on the annotation database used, reflecting variations in database composition, alignment strategies, handling of multi-mapped reads, and length-based filtering parameters. Notably, miRMaster2 detected numerous circRNAs that were not identified by AASRA, whereas AASRA revealed a large number of differentially expressed piRNAs not captured by miRMaster2. piRNAs are well known for their roles in germ cell biology, transposable element silencing, regulation of protein-coding gene expression, and modulation of DNA methylation and histone modification [37]. We previously reported differential expression of several piRNAs in fibroids [18,38]. Similar to miRNAs, piRNAs can bind to the 3′UTR of mRNAs to enhance protein stability and translation or promote mRNA degradation [39,40]. Our current findings indicate that exosomal piRNAs may contribute significantly to fibroid–myometrium signaling, as evidenced by the selective enrichment of piR-1398740 and piR-333378 in Fib-EXO. To date, no published studies have described the function of piRNAs validated here.

Exosomal miRNAs are among the most extensively studied ncRNAs and play major roles as signaling molecules in cancer by regulating proliferation, angiogenesis, metastasis, immune evasion, and chemoresistance [41–43]. They also influence inflammatory responses and myocardial function in cardiovascular disease [44] and contribute to immune modulation within the tumor microenvironment [6,45]. In the present study, we validated expression of several miRNAs with known roles in fibroid pathogenesis. The expression patterns of all PCR-validated miRNAs (miR-200c-3p, miR-29c-3p, miR-133a-3p, miR-490-5p, and miR-105-5p) were concordant with their expression in fibroid tissue with the exception of miR-21-5p, which was not significantly altered in fibroid tissue but was overexpressed in Fib-EXO, suggesting an active process for loading specific cargo within exosomes. Fib-EXO miRNAs may regulate fibrosis, inflammation, and cell proliferation in recipient myometrial, as evidenced by data presented in the current study. Prior work from our group and others implicated the role of miR-29 family in fibrosis [31,46,47], miR-200c in cell cycle regulation and inflammation [48–50], and miR-21 in promoting fibrosis, apoptosis, and proliferation in both cancer and fibroids [34,51–54]. Among the most up-regulated miRNAs in Fib-EXO were miR-490-5p and miR-105-5p, both of which we show here for the first time to be also highly overexpressed in fibroids compared with matched myometrium. These miRNAs are aberrantly expressed in multiple cancers and regulate tumor cell proliferation, apoptosis, and invasion [55,56]. miR-105-5p can function as either an oncogene or tumor suppressor and is detectable in exosomes from several cancer types [56,57].

We also identified numerous differentially expressed exosomal sncRNAs not previously described in fibroids. Among these, the snoRNA, SNORA8 was down-regulated in Fib-EXO and fibroid tissue; snoRNAs primarily regulate RNA processing [58]. Two tRNAs (Gly-GCC-4-1 and Lys-CTT-3-1) were also validated and found to be down-regulated in Fib-EXO, consistent with their reduced expression in fibroid tissue. Full-length tRNA Gly-GCC-4-1 participates in protein translation by delivering glycine to the ribosome. Although the full-length tRNA has been detected in cancer exosomes [59], proliferative effects reported in the literature are attributed to tRF/tiRNA fragments derived from Gly-GCC rather than the mature tRNA itself. Specific Gly-GCC-derived fragments, including tiRNA-Gly-GCC-1 and 5′-tRF-GlyGCC, have been shown to promote proliferation in bladder cancer [60], colorectal cancer [61], and vascular smooth muscle cells [62]. Lys-CTT-3-1, which was also down-regulated in Fib-EXO and fibroid tissue, has been detected in exosomes from various cancers and diseases [63] and has been reported to protect against kidney injury and inhibit ferroptosis [64].

The expression profiles of lncRNAs validated by PCR in Fib-EXO closely mirrored their expression patterns in fibroid tissue reported previously [4,19,30]. Among these, TPTEP1 functions as a tumor suppressor through interactions with miRNAs and proteins and has been shown in several cancers to inhibit PI3K/AKT signaling [65,66]. PART1 exhibits context-dependent behavior, acting as an oncogene in some malignancies and a tumor suppressor in others [67,68]. MSC-AS1, which we found to be down-regulated in Fib-EXO, functions as a molecular sponge for at least 14 miRNAs, including miR-29b-3p, miR-200b-3p, and miR-142-5p, and has been implicated in promoting cell proliferation, invasion, and mesenchymal stem cell differentiation [69]. H19, one of the most highly up-regulated lncRNAs in fibroids [4,19,70], was also abundantly expressed in Fib-EXO. Prior studies have shown that H19 regulates fibroid pathogenesis by sponging let-7 and influencing TET1-mediated DNA demethylation, thereby affecting genes involved in cell proliferation and ECM production [70].

Fib-EXO promoted MSMC proliferation, consistent with RNAseq findings identifying numerous proliferation-associated genes in exosomes, including RPL23, HIPK2, RPS4X, RPS15A, NAMPT, NPM1, TNC, FN1, PTEN, ILK, RPS6, IGF2, CXCL5, SOX4, GAB2, and NAP1L1. Among these, IGF2, which was one of the most overexpressed protein-coding genes in exosomes, stands out as a potent growth factor that is overexpressed in many cancers [71]. IGF2 signals through IGF1R and the insulin receptor A isoform to activate MAPK and PI3K pathways [71]. Its expression is regulated by epigenetic mechanisms, transcription factors such as HMGA2 and PLAG1, and by IGF-binding proteins, particularly IGFBP6, which binds IGF2 and reduces its activity [71,72]. Our data show decreased IGFBP6 in Fib-EXO. RNAseq also revealed reduced levels of ZBED6, a zinc-finger transcription factor known to repress IGF2 expression [73,74]. Together, decreased IGFBP6 and ZBED6 may contribute to elevated IGF2 levels in Fib-EXO.

Similar to cancer-derived exosomes [75,76], Fib-EXO exhibited pro-angiogenic activity. RNAseq identified several angiogenesis-related genes enriched in Fib-EXO, including NCL, CYP1B1, ANXA2, BCAM, CCL2, YWHAZ, FN1, GLUL, SAT1, and ACTG1. The pro-angiogenic effects of Fib-EXO may enhance vascular support for fibroid development, promote neovascularization within the myometrium, and contribute to increased endometrial vascularity. Thus, pro-angiogenic signaling to the myometrium and endometrium by Fib-EXO could potentially represent a mechanism for abnormal uterine bleeding commonly associated with fibroids [77].

A key finding of the present study is the profound impact of Fib-EXO on myometrial gene expression. Fib-EXO were efficiently internalized by MSMCs and induced multiple molecular changes. Treatment of MSMC with Fib-EXO increased vimentin protein levels while reducing COL1A1 and COL3A1, with variable effects on FN1 and α-SMA protein expression. Although the predominant cell type in fibroids is smooth muscle cells rather than epithelial cells, this pattern of gene expression in MSMC treated with Fib-EXO resembles features of epithelial-to-mesenchymal transition (EMT), a process associated with cancer progression and wound healing, characterized by loss of cell–cell adhesion and increased motility [78]. Elevated vimentin expression in smooth muscle cells indicates a shift of smooth muscle cells to a synthetic phenotype that is marked by reduced contractility and enhanced proliferation, migration, and ECM secretion [79,80].

The reduced COL1A1 and COL3A1 expression in MSMCs treated with Fib-EXO was unexpected given the downregulation of collagen-suppressing miR-29 and the increase in TGF-β3, a positive regulator of collagen synthesis [46,47,81]. Because both collagen genes contain promoter CpG islands, an epigenetic mechanism such as promoter hypermethylation may account for this reduction. Increased DNMT1 and EZH2 expression in Fib-EXO-treated MSMCs supports this possibility for epigenetic regulation of collagen. EZH2, the catalytic subunit of PRC2, mediates H3K27 trimethylation and modifies non-histone substrates, regulating cell proliferation, autophagy, and apoptosis [82,83]. EZH2 is up-regulated in many cancers [82,83] and in fibroids [27,84,85]. Another notable gene induced by Fib-EXO was c-MYC, a proto-oncogene overexpressed in fibroids [86] and numerous cancers, where it drives proliferation, apoptosis, metabolism, and differentiation [87]. Other notable genes that were induced by Fib-EXO in MSMC was TGFβ3, a master regulator of fibrosis and highly overexpressed in fibroids where it regulates collagen expression [46,47,81], and the p65 unit of NF-κB. We previously reported that fibroids overexpress the active phosphorylated form of p65 unit of NF-κB [31] and we showed miR-200c, which is down-regulated in Fib-EXO- and MSMC-treated cells, regulates p65 nuclear translocation [49], and Bay 11-7082, an irreversible inhibitor of NF-κB activation, is an effective treatment for fibroid in a mouse model [29]. Together, the induction of c-MYC, EZH2, DNMT1, TGF-β3, and p-p65 suggests that Fib-EXO promote proliferative, inflammatory, and fibrotic reprogramming in MSMCs. Consistent with this, both Fib-EXO and MSMC treated with Fib-EXO showed reduced levels of miR-29-3p, miR-200c-3p, miR-133a-3p and increased levels of miR-21-5p. MIR-105-5p, which was one of the most overexpressed miRNAs in Fib-EXO, was undetectable in MSMC potentially because of very low expression of this miRNA in MSMC. Overall, the genes targeted by these miRNAs regulate proliferation, fibrosis, and inflammation and support the role of exosomal ncRNAs in signaling to target cells.

Only one prior study has addressed the role of fibroid exosomes. In that report, exosomes were isolated from a transformed fibroid cell line that is all smooth muscle cells and lacks immune cells. This study reported similar exosome size as ours. The authors also reported pro-angiogenic effects of fibroid EXO on microvascular endothelial cells. Although small RNAseq was performed, only a miRNA profile was provided without validation of the sequencing data [17]. In contrast with fibroids, more studies exist on adenomyosis, a benign gynecologic disorder with similarities to fibroids and frequent co-occurrence [88]. These investigations have been limited secondary to the use of endometrium-derived rather than adenomyosis-derived exosomes. Nevertheless, these studies also report differential miRNA expression in adenomyosis [89]. Other studies in patients with adenomyosis reported exosome from patients with adenomyosis-induced M2 macrophage polarization via DUSP6/ERK signaling [90], increased proliferation and migration of adenomyotic myometrial cells via IL6/JAK2/STAT3 activation [91], and EMT induction through macrophage polarization [92].

In summary, we provide the first comprehensive transcriptomic and functional characterization of coding and noncoding RNAs in exosomes isolated from primary fibroid and matched myometrial explants, with validation of numerous differentially expressed transcripts in both exosomes and tissue. Fib-EXO are enriched in molecules that regulate cell proliferation, angiogenesis, inflammation, and fibrosis. Functionally, Fib-EXO are internalized by myometrial cells, promoting fibrotic remodeling and a shift of the smooth muscle cells from a contractile to a synthetic phenotype. These findings highlight Fib-EXO as potent mediators of intercellular communication that may drive myometrial proliferation and fibrotic transformation, thereby contributing to fibroid propagation.

## Clinical perspectives

- Background: Uterine fibroids are the most common benign tumors in women and a major cause of abnormal bleeding, pelvic pain, infertility, and hysterectomy. Although fibroids originate from MSMCs, how established tumors modulate the surrounding myometrium to support continued growth remains unclear. Exosomes are critical mediators of intercellular communication, yet their contribution to fibroid pathophysiology has not been defined.
- Results: In the present study, we present the first comprehensive transcriptomic and functional characterization of exosomes derived from primary human fibroids and matched myometrium. Fibroid exosomes exhibited a distinct RNA cargo, including enriched IGF2, H19, PART1, selective piRNAs, and multiple miRNAs linked to proliferation, epigenetic regulation, inflammation, and fibrosis. These vesicles were efficiently internalized by myometrial cells, where they enhanced proliferation, activated pro-fibrotic and pro-inflammatory pathways (EZH2, DNMT1, TGF-β3, c-MYC, p-p65), altered ECM gene expression, and stimulated robust angiogenesis.
- Significance: Overall, Fib-EXO emerge as potent drivers of myometrial reprogramming and represent promising biomarkers and therapeutic targets to interrupt fibroid progression.

## Supplementary Material

Supplementary Table S1

## Data Availability

RNA-seq data have been deposited in GEO under accession number GSE326749 [93]. Derived data supporting the findings of the present study are available from the corresponding author O.K. on request.

## Open Access

Open access for this article was enabled through an open access agreement between the Biochemical Society/Portland Press and the University of California.

## Abbreviations

ECM: extracellular matrix
EMT: epithelial-to-mesenchymal transition
EVs: extracellular vesicles
Fib-EXO: fibroid-derived exosomes
Myo-EXO: Myometrium-derived exosomes
HUVECs: human umbilical vein endothelial cells
lncRNAs: long non-coding RNAs
NGS: next-generation RNA sequencing
PCA: principal component analysis
PPI: protein–protein interaction
sncRNAs: small non-coding RNAs
