# Supplementary material for: Transcriptomic and functional analysis of fibroid extracellular vesicles
Source: Clin Sci (Lond). 2026 May 5;140(5):805–24. doi: 10.1042/CS20250814 (PMC13161198; doi:10.1042/CS20250814)
Supplement: Supplementary Table S1 [file CS-2025-0814_supp.pdf]

Supplementary Table I. Primer sequences used in this study

Primers	Sequences
FBXW2 (Forward)	5'-CCTCGTCTCTAAACAGTGGAATAA-3'
FBXW2 (Reverse)	5'-GCGTCCTGAACAGAATCATCTA-3'
ACTB (Forward)	5'-GGACCTGACTGACTACCTCAT-3'
ACTB (Reverse)	5'-CGTAGCACAGCTTCTCCTTAAT-3'
IGF2 (Forward)	5'-ACCCTCCAGTTCGTCTGT-3'
IGF2 (Reverse)	5'-CGGAAACAGCACTCCTCAA-3'
IGFBP6 (Forward)	5'-AATTCTGCGGGTGTCCAA-3'
IGFBP6 (Reverse)	5'-GGGCACGTAGAGTGTTTGA-3'
HOXA10 (Forward)	5'-GCAAAGAGTGGTCGGAAGAA-3'
HOXA10 (Reverse)	5'-CGCTCTCGAGTAAGGTACATATTG-3'
TPTEP1 (Forward)	5'-CCACAATGGCAGCAAGATTAAG-3'
TPTEP1 (Reverse)	5'-GACGAGAGTGACATCCAGTAAG-3'
PART1 (Forward)	5'-GGCCGTGTCAGAACTCAATTA-3'
PART1 (Reverse)	5'-TATAGCCCAGCGTACGAGAA -3'
MSC-AS1 (Forward)	5'-TAGTTCTAGCATGGTGTGTGTG-3'
MSC-AS1 (Reverse)	5'-TCTCTCTCTCTCTCTCTCTCTCT-3'
H19 (Forward)	5'-CGTGACAAGCAGGACATGA-3'
H19 (Reverse)	5'-TCCGTGGAGGAAGTAAAGAAAC-3'
RNU6-2 (Forward)	5'-CGCTTCGGCAGCACATATAC-3'
RNU6-2 (Reverse)	5'-AGG GGCCATGCTAATCTTCT-3'
miR-490-5p (Forward)	5'-GCAGCCATGGATCTCCAG-3'
miR-490-5p (Reverse)	5'-AGGTCCAGTTTTTTTTTTTTTTTTACC-3'
miR-105-5p (Forward)	5'-TCAAATGCTCAGACTCCTGT-3'
miR-105-5p (Reverse)	5'-GGTCCAGTTTTTTTTTTTTTTTTACCA-3'
miR-21-5p (Forward)	5'-GCAGTAGCTTATCAGACTGATG-3'
miR-21-5p (Reverse)	5'-GGTCCAGTTTTTTTTTTTTTTTTCAAC-3'
miR-29b-3p (Forward)	5'-CAGTAGCACCATTTGAAATCAG-3'
miR-29b-3p (Reverse)	5'-GGTCCAGTTTTTTTTTTTTTTTTAACAC-3'
miR-29c-3p (Forward)	5'-GCAGTAGCACCATTTGAAATC-3'
miR-29c-3p (Reverse)	5'-GGTCCAGTTTTTTTTTTTTTTTTAACC-3'
miR-200c-3p (Forward)	5'-AGTAATACTGCCGGGTAATG-3'
miR-200c-3p (Reverse)	5'-GTCCAGTTTTTTTTTTTTTTTTCCATC-3'
miR-133a-3p (Forward)	5'-TGGTCCCCTTCAACCAG-3'
miR-133a-3p (Reverse)	5'-GGTCCAGTTTTTTTTTTTTTTTTCAG-3'
miR-423-5p (Forward)	5'-CAGTGAGGGGCAGAGAG-3'
miR-423-5p (Reverse)	5'-GGTCCAGTTTTTTTTTTTTTTTTAAAGTC-3'
piR-1398740 (Forward)	5'-GCATTGTAGGTTCTGGATATTAGC-3'
piR-1398740 (Reverse)	5'-GGTCCAGTTTTTTTTTTTTTTTTAAAGG-3'
piR-333378 (Forward)	5'-GCAGTTGGAGAAAACAGAAAATTC-3'
piR-333378 (Reverse)	5'-GGTCCAGTTTTTTTTTTTTTTTTACTCA-3'
piR-137586 (Forward)	5'-TGGGTGGTTCAGTGGTAG-3'
piR-137586 (Reverse)	5'-GGTCCAGTTTTTTTTTTTTTTTTCTG-3'
piR-174142 (Forward)	5'-CCTGGTGGTCTAGTGGTTAG-3'
piR-174142 (Reverse)	5'-GTTTTTTTTTTTTTTTGGCGCCGT-3'
Gly-GCC-4-1 (Forward)	5'-GCATAGGTGGTTCAGTGGTAG-3'
Gly-GCC-4-1 (Reverse)	5'-GTCCAGTTTTTTTTTTTTTTTGCAT-3'
Lys-CTT-3-1 (Forward)	5'-CTCAGTCGGTAGAGCATGAGACCCTTAATCTCAGGGTCGTG-3'
Lys-CTT-3-1 (Reverse)	5'-GTTTTTTTTTTTTTTTCGCCCAAC-3'
SNORA8 (Forward)	5'-GGTATCTGCACTCAGCAGTTTA-3'
SNORA8 (Reverse)	5'-AACAAGCACAAAGCCAAGAAA-3'
